# Endoscopic submucosal dissection of Barrett’s neoplasia using adaptive multipolar traction combined with line: report of two cases

**DOI:** 10.1055/a-2217-3544

**Published:** 2024-01-09

**Authors:** Jean Grimaldi, Louis-Jean Masgnaux, Alexandru Lupu, Timothée Wallenhorst, Jérôme Rivory, Jérémie Jacques, Mathieu Pioche

**Affiliations:** 1 36609 Gastroenterology and Endoscopy Unit, Hopital Edouard Herriot, Lyon, France; 2 36684 Endoscopy and Gastroenterology, Centre Hospitalier Universitaire de Rennes, Rennes, France; 3 37925 Hepatogastroenterology, Centre Hospitalier Universitaire Dupuytren, Limoges, France

Endoscopic submucosal dissection (ESD) is the gold standard for the resection of visible lesions of >15 mm in Barrett's neoplasia 1 . Recently, the “tunnel + traction with a line” strategy has shown encouraging results in terms of safety and efficacy 2 3 . However, the traction with a line technique has the disadvantage of exerting a traction force that is quasi-tangential to the plane of the submucosa. We believed that combining this with the ATRACT 2+2 adaptive multitraction device ( Fig. 1 ) 4 5 would enable a 90° traction force to be exerted, which could also be increased during the procedure.

**Fig. 1:**
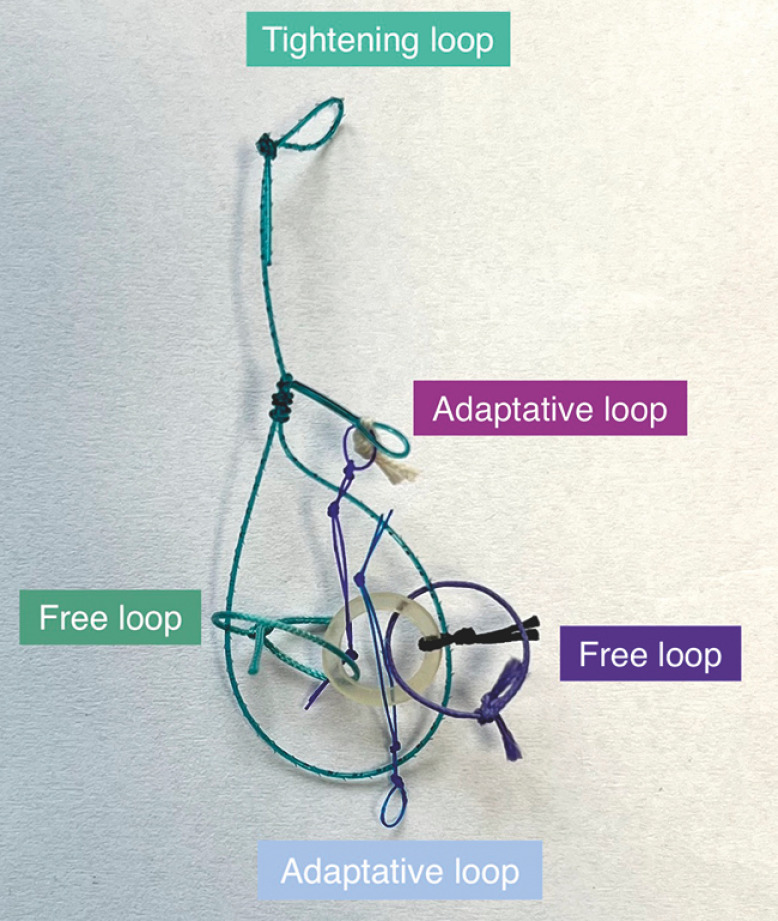
Photograph of the ATRACT 2+2 device.

We report here on two patients referred for ESD resection of Barrett esophagus (BE), using the adaptive multipolar traction combined with a line ( Video 1 ).

Endoscopic submucosal dissection of Barrett’s neoplasia using adaptive multipolar traction combined with a line.Video 1

The first patient presented with a Barrettʼs neoplasia within a pseudodiverticular hiatal hernia. After a circumferential incision had been made at the distal pole, two tunnels (anterior and posterior) were made. The ATRACT 2+2 device attached to a line was then placed with three points on the proximal edge and one point on the distal edge, so as to obtain an optimum 90° angle of traction. The line attached to the ATRACT 2+2 device prevents the lesion from tilting towards the stomach during the procedure. After two-thirds of the dissection had been completed, the ATRACT 2+2 device was tightened to increase the traction ( Fig. 2 ) and facilitate completion of the procedure ( Fig. 3 ).

**Fig. 2:**
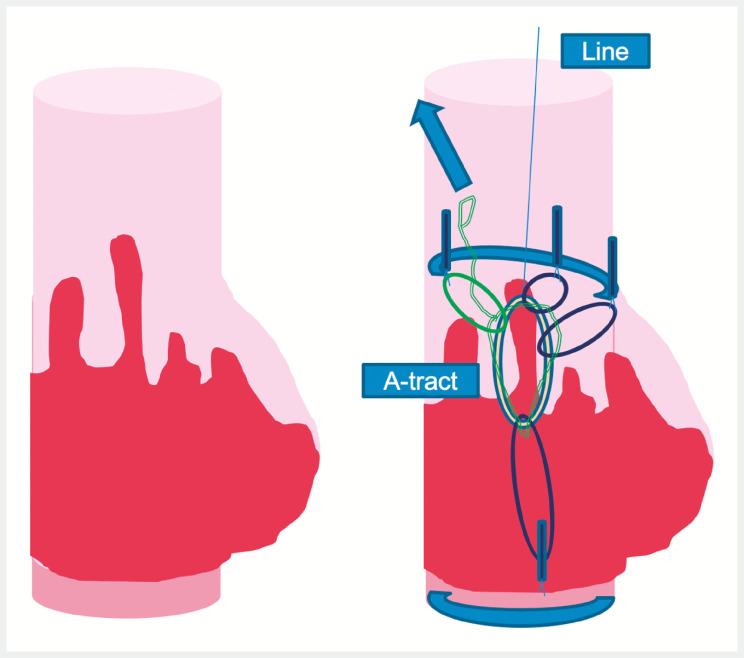
Schematic representation of the use of multipolar adaptive traction combined with a line.

**Fig. 3:**
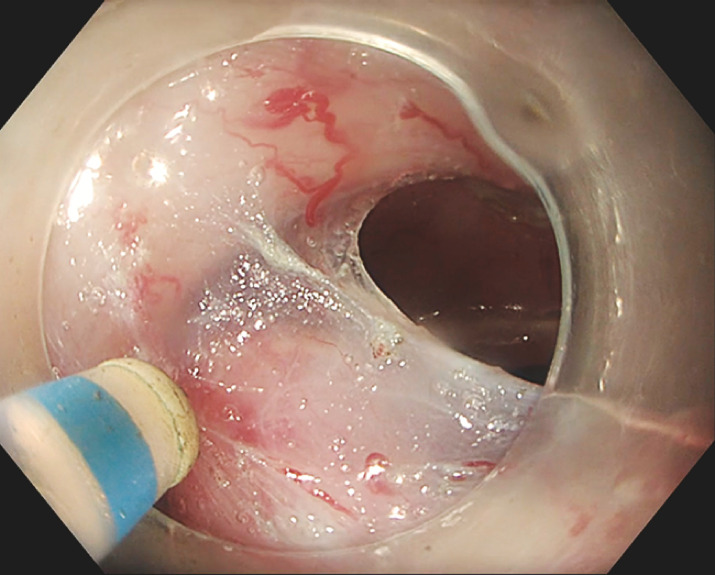
Endoscopic view showing the optimal 90° traction angle being maintained at the end of the endoscopic submucosal dissection procedure when the ATRACT 2+2 device is used with a line.

The second patient underwent a near-circumferential BE dissection, using a similar strategy. The two procedures were carried out by two different operators.

The dissections enabled en bloc resection of both lesions, the first corresponding to an intramucosal adenocarcinoma and the second to a T1b sm2 adenocarcinoma. There were no complications, in particular no perforations, during the procedures.

Further investigations are needed to evaluate the efficacy of the multipolar adaptive traction device combined with a clip-and-line traction strategy for the resection of esophageal lesions, but the technique seems promising.

Endoscopy_UCTN_Code_TTT_1AO_2AG

## Notes

Endoscopy E-Videos https://eref.thieme.de/e-videos
*E-Videos* is an open access online section of the journal *Endoscopy* , reporting on interesting cases and new techniques in gastroenterological endoscopy. All papers include a high-quality video and are published with a Creative Commons CC-BY license. Endoscopy E-Videos qualify for HINARI discounts and waivers and eligibility is automatically checked during the submission process. We grant 100% waivers to articles whose corresponding authors are based in Group A countries and 50% waivers to those who are based in Group B countries as classified by Research4Life (see: https://www.research4life.org/access/eligibility/ ). This section has its own submission website at https://mc.manuscriptcentral.com/e-videos .
